# Biomimetic Hydroxyapatite Crystals Growth on Phosphorylated Chitosan Films by In Vitro Mineralization Used as Dental Substitute Materials

**DOI:** 10.3390/polym15112470

**Published:** 2023-05-26

**Authors:** Fathia Rahmani, Omar Larbi Bouamrane, Amina Ben Bouabdallah, Leonard I. Atanase, Abdelkader Hellal, Aurelian Nichita Apintiliesei

**Affiliations:** 1 Department of Technology, Faculty of Science and Technology, University of Djillali Bounaama, Theniet El Had Street, Khemis-Miliana, Ain Defla 44225, Algeria; f.rahmani@univ-boumerdes.dz; 2 Laboratory for the Processing and Shaping of Fibrous Polymers (LTMFP), M’Hamed Bougara University, Boumerdes 35000, Algeria; ben-bouabdallah@univ-boumerdes.dz; 3 Institute of Science, University Center of Tipaza Morseli Abdallah, Oued Merzoug, Tipaza 42022, Algeria; 4 Laboratory of Natural Substances Valorization (LVSN), Faculty of Science and Technology, University of Djillali Bounaama, Theniet El Had Street, Khemis-Miliana, Ain Defla 44225, Algeria; haekpharm@yahoo.fr; 5 Department of Process Engineering, Faculty of Technology, University of M’hamed Bougara, Boumerdes 35000, Algeria; 6 Food Technology Laboratory, University of M’hamed Bougara, Boumerdes 35000, Algeria; 7 Faculty of Medical Dentistry, “Apollonia” University of Iasi, 700511 Iasi, Romania; dr.apintiliesei@yahoo.com; 8 Academy of Romanian Scientists, 050045 Bucharest, Romania; 9 Chemistry Department, Faculty of Sciences, University of Ferhat Abbas-Sétif-1, Sétif 19137, Algeria

**Keywords:** phosphorylated chitosan, chitosan film, biomineralization, calcium phosphate, hydroxyapatite, dental material

## Abstract

Chitosan (CS) films exhibit great potential as a substrate for the in vitro mineralization process. In this study, to mimic the formation of nanohydroxyapatite (HAP) as natural tissue, CS films coated with a porous calcium phosphate were investigated using scanning electron microscopy (SEM), Energy dispersive X-ray spectroscopy (EDX), Fourier transforms infrared spectroscopy (FTIR), X-ray diffractometry (XRD) and X-ray photoelectron spectroscopy (XPS). Calcium phosphate coating deposited on phosphorylated derivatives of CS was obtained by a process based on phosphorylation, Ca(OH)_2_ treatment and artificial saliva solution (ASS) immersion. The phosphorylated CS films (PCS) were obtained by partial hydrolysis of the PO_4_ functionalities. It was demonstrated that this precursor phase could induce the growth and the nucleation of the porous calcium phosphate coating when immersed in ASS. Moreover, oriented crystals and qualitative control of calcium phosphate phases on CS matrices are obtained in a biomimetic mode. Furthermore, in vitro antimicrobial activity of PCS was evaluated against three species of oral bacteria and fungi. It revealed an increase in antimicrobial activity with minimum inhibition concentration (MIC) values of 0.10% (*Candida albicans*), 0.05% (*Staphylococcus aureus)* and 0.025% (*Escherichia coli*) which proves their possible use as dental substitute materials.

## 1. Introduction

One of the first uses of artificial biomaterials was in the field of dentistry for the treatment or replacement of different diseased tissues. Dentistry has benefited from the progress in biomaterials and biomedical devices over the last decades. Numerous studies investigated synthesising and characterising new biomacromolecules and complex biocompatible materials for dental clinical applications. As each biomolecule has a specific set of properties, it is essential to explore and set up the right procedure to obtain a complex biomaterial with features from all the individual components [1].

Complex biological nanocomposites, such as human bone and teeth, contain nanocrystalline apatite minerals implanted in a three-dimensional matrix of protein collagen fibrils. Calcium phosphates have a proportion of 60 to 70% in bone, whereas a higher amount of 97% is found in dental enamel. Calcium phosphate is carbonate-substituted hydroxyapatite with percentages between 2.3 and 8 wt.% [2,3]. Dental diseases are recognized as one of the significant and most common diseases afflicting mankind throughout the world [4]. Annually, many humans are affected by bone defects induced by trauma, tumor or bone diseases and need orthopedic or dental/craniofacial implants and bone grafting biomaterials [5,6].

Biomaterials are a branch of materials with important advantages in dentistry and orthopedic applications. For example, to repair a diseased or damaged part of hard tissue, such as bone, hips, knees and teeth, bone-substituted biomaterials are necessary [7].

The preparation of bio-inspired or bio-mimetic composites is one of the recent research fields, as these materials can be used in high-value applications. The presence of such complex and highly ordered materials, based on a mixture of a polymer matrix reinforced with inorganic crystallites, is ubiquitous, and some specific examples are bone, dentine, eggshell, and the shell of marine mollusks. These composite materials have a similar hierarchical structure defined over several length scale levels [8,9]. Furthermore, these materials’ micro and macroscopic properties are further influenced by their composition and structure, which can be controlled by biomimetic approaches (for the structure) and chemistry (for the composition).

Intelligent biomaterials used for tissue engineering applications are, at present, obtained from polymer-based composites, which are dispersed nanocrystallites of calcium phosphate (CaP) salts [10]. The coating of different types of biomaterials can be carried out by the biomimetic deposition of CaP from simulated body fluid [11,12]. For example, to increase osteogenesis and implant fixation, the surface of implants was modified using CaP due to a similar structure of the biomimetic CaP to that of the natural bone minerals [13]. An additional advantage of this method is that osteogenesis and wound healing can be promoted by coating-immobilized growth factors or antibiotics [14].

Today, one of the most challenging works is the design of mineralized biopolymer-based materials for modern biomedical applications. In this context, chitosan (CS), the fully or partially deacetylated form of chitin, has already proved its potential as a highly efficient biopolymer. The non-toxic and enzymatically biodegradable CS is the principal component of living organisms, such as fungi and crustaceans, containing 2-acetamido-2-deox-d-glucopyranose and 2-amino-2-deoxy-β-d-glucopyranose groups. Due to these properties and its antimicrobial activity, mucoadhesive, cell adhesion and migration, CS is frequently used in biomedical applications [15,16,17,18,19,20]. Moreover, the polycationic nature of CS in acidic solutions is provided by the presence of free amino groups within its structure. In addition, the chemical modifications of CS are feasible through its amino and hydroxyl groups. The newly obtained bifunctional material will retain the original physicochemical and biochemical properties of the CS skeleton. However, the groups introduced will bring on specific properties in function of their nature. Recently, phosphorylated derivatives of CS, owing to their properties, were obtained by different techniques [21,22,23]. Moreover, these modifications, using phosphonic acid or phosphonate, modified the solubility of CS.

Recent studies on bone regeneration are based on preparing porous materials that can be employed as scaffolds for tissue regeneration or replacement in a way very similar to the natural one. To exploit the favourable properties of modified CS, composite CS and biomimetic calcium phosphate materials were investigated for their possible usage in different domains, such as implant coatings, tissue engineering scaffolds, films, cements, and composites [24,25,26,27,28,29,30,31,32]. These studies conclude that bio-mimetic CaP (hydroxyapatite) (HAP) could be deposited on phosphorylated CS (PCS) after immersion in 1 and 1.5 SBF solutions for 30 days. However, to the best of our knowledge, no results are reported on the role of phosphorylated derivatives of CS on the preparation and characterization of bio-mimetic coatings in dental applications as remineralized and substitute materials.

This study investigated the technique for obtaining ideal local conditions for the nucleation and growth of HAP nanocrystals on CS films. This technique is based on surface modification followed by immersion in artificial saliva solution (ASS). Based on the above considerations, to control the growth and nucleation of HAP in a bio-mimetic root, PCS was prepared and used to initiate osteogenesis when implanted in dental sites.

Modifying the substrate with phosphorus–containing groups was very efficient for the CaP growth on PCS films using the urea/H_3_PO_4_ method followed by immersion, during one week, in saturated Ca(OH)_2_ solution and ASS for various durations. The novel bio-mimetic CS nanohydroxyapatite composite was investigated by Fourier transform infrared spectroscopy (FTIR), X-ray diffraction (XRD), X-ray photoelectron spectroscopy (XPS), Inductively Coupled Plasma analysis (ICP) and scanning electron microscopy (SEM).

## 2. Materials and Methods

### 2.1. Materials

CS (Mw 1450 kDa, deacetylation degree (DD) 80.79%) was purchased from FLUKA, Germany. The DD of the CS sample used in this experiment was obtained by the FTIR method [33], and viscosity molecular mass was calculated using an Ubbelhode viscosimeter as previously described [34]. No purification was carried out for all other compounds.

### 2.2. Preparation of CS Films

CS films were obtained by dissolving 1 g of CS powder in 100 mL acetic acid solution at a concentration of 1 wt%. The solution was stirred for 6 h to allow the full dissolution of CS and then poured into round glass Petri dishes. The obtained films were dried at room temperature. After three days, they were carefully removed and quickly neutralized by rinsing with saturated NaOH solution and then washed with distilled water. After drying overnight at room temperature, films were cut into 1 cm^2^ rectangular shapes.

### 2.3. Biomimetic Mineralization of PCS Films

To obtain the biomimetic CaP coating, the CS films were first phosphorylated to create functional sites on their surface that will further facilitate the nucleation and growth of nanocrystals. The phosphorylation was carried out by a similar procedure adapted from Varma et al. [24]. In a flask fitted with a thermometer, condenser and N_2_ gas inlet tube mixed 1 g of CS films, 15 g of urea, 3 g of 98% H_3_PO_4_ and 30 mL of *N,N*-dimethyl formamide. The flask with the reactants was then heated to 120 °C for one hour under gentle stirring. After cooling under flowing N_2_ gas, the films were thoroughly rinsed with distilled water.

The phosphorylated films were immersed in a saturated Ca(OH)_2_ solution at 37 °C for eight days. The Ca(OH)_2_ solution was renewed every four days. After treatments, the CS films were thoroughly washed with distilled water. This procedure has, as a consequence, the development of CaP precursor sites for the growth of HAP nanocrystals.

After filling with Ca(OH)_2_ soaking, the samples were freely suspended vertically in plastic flasks to which 50 mL of ASS was added. The flasks were then covered and put into an oven at 37 °C. The ASS was renewed daily, and films were removed after 4, 15 and 28 days of biomineralization. Then, distilled water was used to wash the films, allowing them to dry at room temperature.

Many formulations of ASS have been described to simulate natural saliva with an electrolyte composition similar to human saliva [35,36,37,38]. ASS used in this study was prepared as previously described [36] and had the following composition: 0.1 L each of 25 mM K_2_HPO_4_, 24 mM Na_2_HPO_4_, 150 mM KHCO_3_, 100 mM NaCl, and 1.5 mM MgCl_2_. To this were added 0.006 L of 25 mM citric acid and 0.1 L of 15 mM CaCl_2_. A total volume of 1 L was obtained, and the pH was adjusted to 6.8–7.0 with NaOH or HCl at 37 °C. Finally, 0.05% by weight thymol was added to the artificial saliva to avoid bacterial growth. The solution was stable for at least 20 weeks in a closed plastic bottle. The final composition of ASS is given in Table 1 and compared to the composition of human saliva.

### 2.4. Characterization of Biomimetic Mineralized Films

Scanning electron microscopy SEM and EDX analysis was carried out using a (Philips Quanta 200) scanning electron microscope equipped with an energy-dispersive X-ray spectroscopy (EDX) microanalysis system for the surface morphology examination of the biomimetic mineralized films and the Ca/P ratio determination of the coated CS films.

The presence of crystalline phases in the biomimetic coating was observed by X-ray diffraction (XRD) using a (PHILIPS Panalytical) X-ray diffractometer.

FTIR spectra were automatically recorded using a transparent KBr matrix (FTIR-800, Shimadzu spectrometer) between 400–4000 cm^−1^ in KBr pellets. The number of scans was ten, and the resolution was 16 cm^−1^.

The XPS tests were carried out using a Kratos Axis Ultra Spectrometer (Kratos Analytical, Manchester, UK) equipped with a monochromatizedaluminium X-ray source (powered at 10 mA and 15 kV) and an eight channeltrons detector. Additional information can be found in the Supporting Information section.

### 2.5. Weight Test

Weighed dry films were soaked in ASS at room temperature, and then at fixed times, the films were removed, the excess solution was blotted from the surface with filter paper, and then weighed. The weight variation of the films (W%) versus the time of biomineralization in ASS solution was determined by (Equation (1)), where M_d_ and M_s_ represent the mass of dry and swollen films, respectively.

(1) $$W\left(\%\right)=\frac{M_{S}-M_{d}}{M_{d}}100$$

### 2.6. Evaluation of Antimicrobial Activity of PCS In Vitro

Minimum inhibitory concentration (MIC) is the lowest concentration that ultimately inhibits the growth of bacteria compared with the control, disregarding a single colony or a faint haze caused by the inoculum [39].

The liquid dilutions method was used to determine MIC values of PCS following the procedure described in the literature [38,39] with slight modifications. A 1% (*w*/*v*) solution of PCS was prepared in 1% (*v*/*v*) acetic acid. Duplicate two-fold serial dilutions of each sample were added to Soga liquid nutrient medium for final concentrations of (0.00625, 0.0125, 0.025, 0.05, 0.10 and 0.15%). As a control, acetic acid was used at 0.1% (*v*/*v*). All samples were autoclaved at 121 °C for 25 min. Each bacterium culture was diluted to 10^5^ CFU/mL with sterile distilled water. The sample with each suspension was inoculated on a nutrient medium. And were then incubated for 24 h at 37 °C. The Minimum Inhibitory Concentration values were obtained after the colonies were counted.

## 3. Results and Discussion

The original CS films and the phosphorylated ones were transparent, and the surface of both samples appeared smooth by SEM. As per the XPS analyses, phosphor (P) content in the phosphorylated chitosan films was 1.52%.

### 3.1. FTIR Results

**Figure 1:**
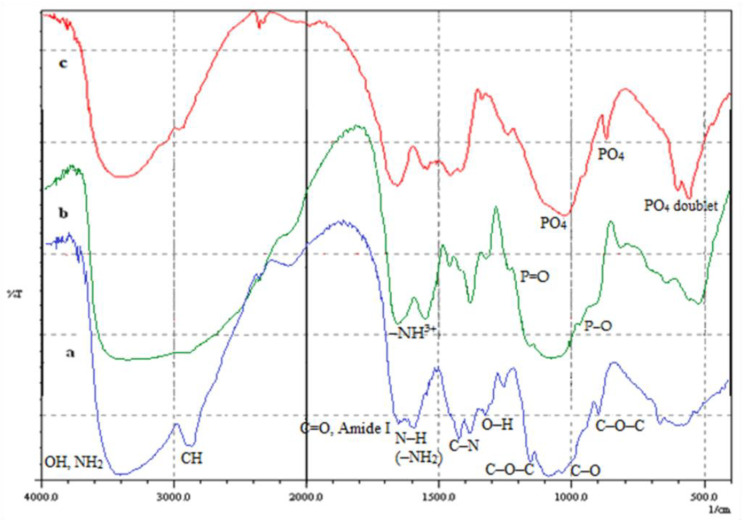
FTIR spectrum of (a) CS film, (b) PCS film and (c) Ca(OH)_2_ treated PCS film.

FTIR spectra of unmodified and phosphorylated CS films are illustrated in Figure 1. The spectrum of unmodified CS films displays specific peaks of amide I from acetylated amine groups at 1654 cm^−1^ (C=O stretching). The peak at 1595 cm^−1^ is assigned to N−H deformation in the amino group. This peak has a strong intensity and covers up the peak of amide II at 1545 cm^−1^ [21,29]. The peak at 1419 cm^−1^ is assigned to the C−N stretching of −C−NH_2_ [21]. The peak at 1033 cm^−1^ is assigned to the C−O stretching of −CH_2_−OH, whereas the peak at 1070 cm^−1^ is assigned to the C−O stretching of −CH−OH. The peak at 1153 cm^−1^ is the (C−O−C) stretching [21,29,40]. Peaks at 1380 cm^−1^ and 1323 cm^−1^ are attributed to O−H deformation of −CH−OH and −CH_2_−OH [21]. The broad peak at 3200–3450 cm^−1^ is attributed to the hydrogen-bonded OH stretching at 3425 cm^−1^ and the NH_2_ asymmetric stretching at 3367 cm^−1^, whereas the peak at 2883 cm^−1^ is assigned to CH stretching [29,40].

For phosphorylated chitosan films (PCS), the emerging shoulder peak at 1210 cm^−1^ is assigned to the P=O stretching [21,29,41]. Similar observations were also obtained for phosphorylated chitin [24]. The peak at 972 cm^−1^ is attributed to P−O stretching [29,41]. In addition, the peaks of the N−H deformation of the amino group at 1595 cm^−1^ and the C−N stretching at 1419 cm^−1^ decrease in intensity, which indicates that the amino groups contributed to the reaction of phosphorylation. It can indicate that a chemical bond appears between the phosphate and amino groups rather than an ionic bond. That is to say. The phosphate group substitute one hydrogen atom of the amino group to form N−P bond [21]. In this phosphorylation reaction, the only reactive functional group is the amino one; this can be confirmed by the fact that the peaks at 1380 cm^−1^ and 1323 cm^−1,^ characteristic of hydroxyl groups, showed no change or shift. In addition, the peak at 1595 cm^−1,^ attributed to the N−H deformation, still exists. Only a part of the amino groups participated in the reaction of phosphorylation [21,29,31]. The anti-symmetrical deformation at 1651 cm^−1^ and the symmetric deformation at 1557 cm^−1^, both attributed to the −NH^3+^ cation, demonstrate the protonation of CS amino groups. These vibrations can overlap the initial amide I and II bands [42].

After the eight-day immersion of PCS films in Ca(OH)_2_ solution, their surface was covered with fine particles. It was determined to be calcium phosphate obtained by partial hydrolysis of the PO_4_ functionalities. These particles act as nucleation layers for further CaP deposition when the film is immersed in ASS. In addition to the covalently bound phosphates, the present phosphorylated films have phosphate groups ionically connected to protonated CS amine functionalities. These ions significantly contribute to the anchorage of calcium ions and, thus, to the apparition of CaP precursor sites.

The XPS analysis calculated a Ca/P ratio of 2.33, which indicates a large amount of calcium in the sample, attributed to the presence of Ca(OH)_2._ In addition, it was demonstrated that CS phosphate is a very good cation absorber [26,28,29].

At pH > 6.3, octacalcium phosphate (OCP) is the favorite precursor for supersaturation in solutions containing Ca^2+^ and HPO_4_^2−^ ions. Moreover, it might be possible that Ca(OH)_2_ has partially hydrolysed the CS-PO_4_ groups to produce, in an initial phase, OCP, which is then quickly converted into calcium-deficient apatite [25,26,28].

Octacalcium phosphate (Ca_8_H_2_(PO_4_)_6_·5H_2_O), the prototype in bones and teeth apatite crystals, was reported to be a precursor of biological apatite crystals due to its structural relation to HAP. After the conversion of OCP, the morphologic and structural features of HAP crystals are similar to bone apatite crystals regarding the plate-like morphology carbonate substitution in the phosphate position and Ca deficiency of the crystals [43,44]. It was shown in the literature that this mineral phase enhances bone formation to a higher extent than synthetic HAP if implanted in bone defects during conversion from OCP to HAP [43,44,45]. The formation of OCP can be described by the reaction: 8Ca^2+^ + 6HPO_4_^−^ + 5H_2_O→Ca_8_H_2_(PO_4_)_6_·5H_2_O + 4H^+^.

Figure 1c revealed a poorly crystalline calcium phosphate layer. Moreover, the spectra of these samples show specific peaks of phosphate vibrations in apatitic structures overlaying those of the PCS. The intense band at 1029.9 cm^−1^ was assigned to the PO_4_ vibration, the peak at 960 cm^−1^ was attributed to the PO_4_ vibration, and the peaks at 604 and 563 cm^−1^ to the PO_4_ vibration can be attributed to the OCP structure [44]. In the FTIR spectrum, the doublets of PO_4_ (604, 563) cm^−1^ indicate that the precursor phase of the HAP formed was OCP and not amorphous calcium phosphate (ACP). When HAP is formed from ACP, the band is a broad singlet, whereas in the case of OCP, band splitting usually occurs, indicating that the minerals formed were poorly crystalline but not amorphous [30,46].

**Figure 2:**
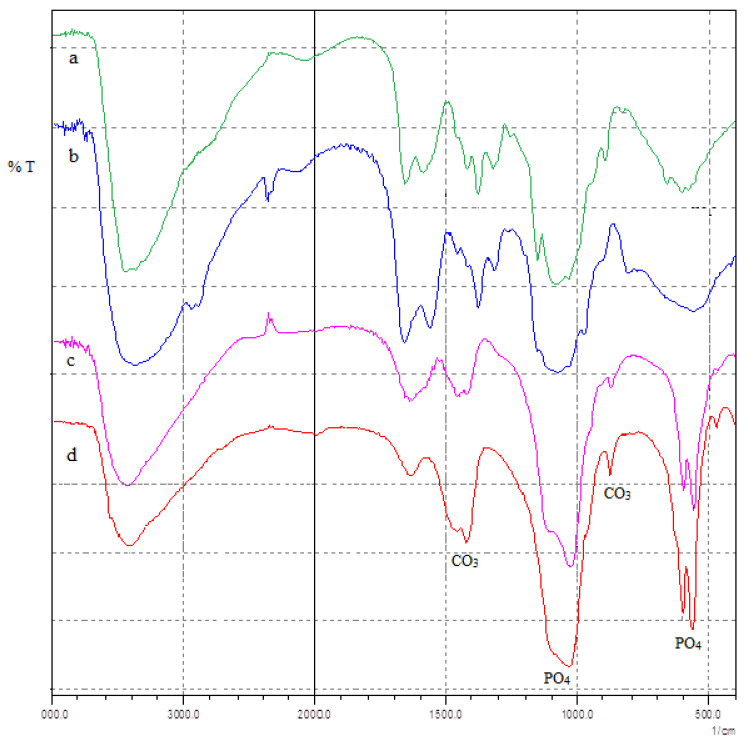
FTIR spectra of Ca(OH)_2_ treated phosphorylated chitosan film after soaking in ASS for (a) 4 days, (b) 15 days, (c) 21 days and (d) 28 days.

Figure 2 shows the spectra obtained for the different Ca(OH)_2_ treated PCS films after 4, 15, 21 and 28 days of biomineralization in ASS.

The FTIR spectrum illustrates that two distinct bands at 1195 and 916 cm^−1^ (assignable to HPO_4_^2−^ ions in the OCP configuration) are visible for OCP but not apatite (Figure 2c). These differences may be considered as other elements for differentiation between these two phases [47].

It can be assumed that the thin coatings of CaP material on the films, produced by partial hydrolysis of the CS-PO_4_ saturated Ca(OH)_2_ functionalities during soaking in solution, act as a nucleation layer for the growth of CaP from the ASS solution. In general, it was observed that the crystal growth starts immediately after one day of immersion.

The partial hydrolysis of OCP to HAP is induced by the higher solubility of OCP (pK ≈ 99) compared to that of HAP (pK ≈ 118). This is followed by a re-precipitation of HAP in a Ca^2+^ containing solution as described in reaction [42]: Ca_8_H_2_(PO_4_)_6_·5H_2_O + 2 Ca^2+^→Ca_10_(PO_4_)_6_(OH)_2_ + 3 H_2_O + 4 H^+^.

In this reaction, the calcium ions are consumed. In contrast, phosphate ions are released into the solution as demonstrated by in vitro tests which showed that Ca^2+^ diffused into and PO_4_^3−^ out of the OCP lattice during HAP formation [44].

**Figure 3:**
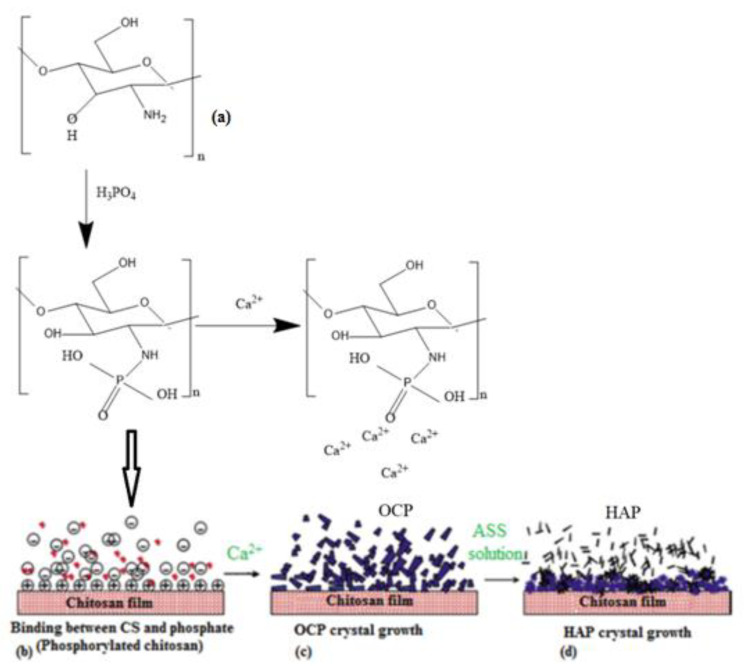
Schematic illustration of the mineralization on PCS film. (**a**) Scheme of phosphorylated and mineralized CS, (**b**) Interaction between CS and phosphate to form phosphorylated chitosan films, (**c**) Formation of OCP phase after addition of Ca^2+^ ions, and (**d**) HAP crystals grow.

The overgrowth mechanism of apatite around the plate-like OCP template during initial enamel mineral formation has been proposed (Figure 3). OCP can be also transformed into HAP via a dissolution–reprecipitation mechanism. Consequently, this reaction has the morphology change of OCP, and it is based on the exchange of calcium and phosphate ions between OCP and tissue fluid during the formation of a new bone [43].

Intensity band attributed to the (HPO_4_)^2−^ [P−(OH)] stretching decreased due to the hydrolysis of OCP to HAP, which occurred during the immersion in ASS. Moreover, the broadband, in the range 960–1200 cm^−1^, can be assigned to the P−O asymmetric stretching mode of the (PO_4_)^3−^ group illustrating the deviation of the phosphate group from the ideal tetrahedral structure [44].

By increasing the immersion duration in ASS, the OCP precursor became more apatitic. This behavior is demonstrated by the degradation of the central 1075 cm^−1^ absorption band and the appearance of a shoulder. Peaks were observed at 871 cm^−1^, 1419 cm^−1^ and 1460 cm^−1,^ and they were assigned to the vibration of carbonate groups (CO_3_), indicating the existence of carbonated apatite, usually found in carbonate and biological apatites [26,30,44,47]. The high Ca/P ratio is a consequence of the presence of carbonate groups.

Apatite is formed in OCP during the immersion in ASS, and this behavior is confirmed by a sharp rise in the intensity of CO_3_ bands (1460 and 1419 cm^−1^) and by a shift of the 859 cm^−1^ peak to 876 cm^−1^. The main signal of phosphate appears in the domain (1000–1100 cm^−1^), and a doublet appears in the region of the 1162 cm^−1^ band (Figure 2d). This mechanism is identical to the self-organization processes of the collagen–apatite bone tissue matrix during biomineralization in vivo [48]. The intense peak at 1026 cm^−1^ was attributed to the PO_4_ vibration. Moreover, the presence of the distinct peaks at 469, 559 and 601 cm^−1^ are assigned to the symmetric P−O stretching vibration of PO_4_^3−^ confirming thus the formation of hexagonal hydroxyapatite. Hydroxyl stretching of −OH due to an organized water structure in HAP appears at 3421 cm^−1^. The absence of the C−O absorption bands at 700 cm^−1^ proves the lack of calcite associated with HAP. OH^−^ or PO_4_^3−^ ions (type A CO_3_^2−^ or type B CO_3_^2−^) can be substituted by carbonate ions in the apatite structure [49].

### 3.2. XRD Results

XRD was used, in this study, to identify the mineral phase and to confirm the change in the crystallinity degree of unmodified CS and PCS films, as well as films after bio-mineralization in ASS. Figure S1 illustrates the XRD spectra of the unmodified CS and PCS. The presence of broad peaks between 20° and 30° shows the semi-crystalline nature of CS, which is formed by both amorphous and crystalline phases. Similar characteristic of chitosan and phosphorylated chitosan was obtained in previous research [50]. Moreover, the crystalline structure of PCS is compared with that of CS, and it can be observed that the peaks of PCS at 20° (110) (Figure S1b) were less intense than that of CS, indicating the presence of a phosphorous group into CS. The introduction of phosphate groups disturbed the ordered structure of CS. It led to weaker hydrogen-bonding interactions between chains in PCS which had, as a consequence, a decrease in crystallinity [51].

**Figure 4:**
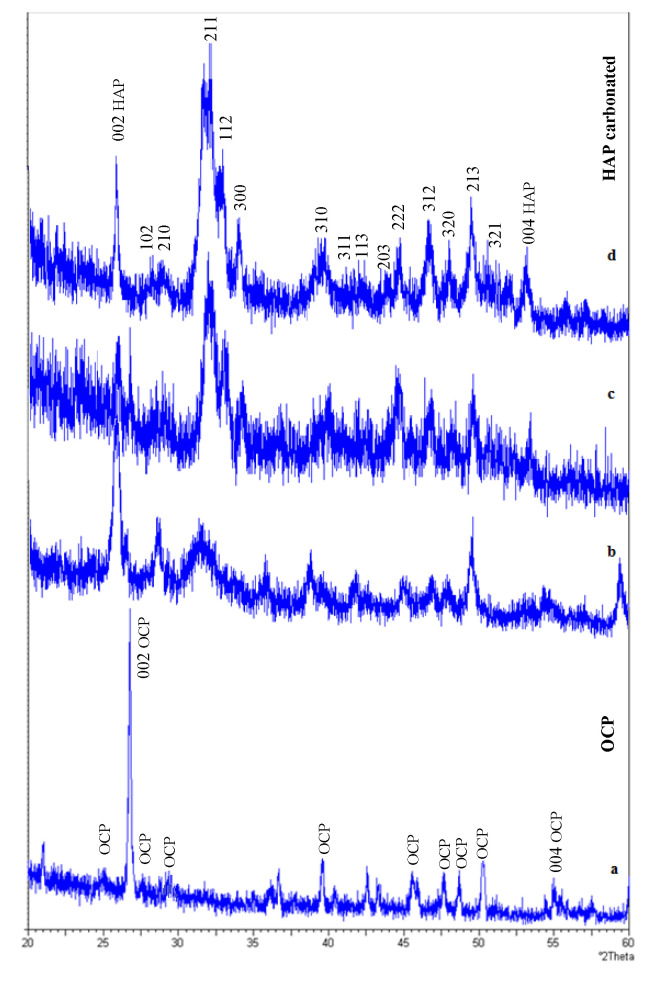
XRD spectra of samples of (a) Ca(OH)_2-_treated PCS films. Soaking time in ASS: (b) 4 days, (c) 15 days and (d) 28 days.

For Ca(OH)_2_ treated PCS films (Figure 4a), a diffraction peak at approximately 26° was found and could be attributed to the (002) reflection of OCP.

As observed in Figure 4, both OCP and HAP contribute to the diffraction peak at 26°. However, it can be noticed that, according to the powder diffraction file cards of OCP (No. 00-026-1056) and HAP (No. 00-009-0432), the diffraction angle corresponding to the OCP (002) reflection is slightly higher than that of the HAP (002). The same conclusions were obtained by Ohtsu et al. [52]. Moreover, XRD tests show that this peak had a symmetric shape, indicating the absence of overlapping with other peaks. On the contrary, the diffraction angle of the Ca(OH)_2_ treated PCS films was slightly higher than that of the bio-mineralized films. For these films, it appears that the OCP (002) reflection peak is high, meaning that the c-axis of OCP is oriented perpendicular to the substrate surface [52]. The absence of the reflection (211) plane in the X-ray pattern of the Ca(OH)_2_ treated PCS sample proves that the HAP phase did not form during OCP precipitation [53].

X-ray patterns, given in Figure 4, show that the peak intensity at around 2θ = 20° decreases sharply by increasing the immersion time in ASS; meanwhile, the crystallinity of the biomineralized films became higher. In Figure 4b–d, compared with the spectrum of HAP powder, the spectrum of biomineralized films in ASS for 4, 15 and 28 days lacked a specific diffraction pattern of crystalline HAP. The XRD patterns reveal the presence of main characteristic peaks of HAP crystals at 26° for reflection (002) and triplet at 32° consisting of reflections from (211) at 31.8°, (112) at 32.2° and (300) at 33.6°. After soaking for 28 days in ASS, a clearer spectrum is observed for the analyzed samples (Figure 4d), indicating a crystalline HAP, (210), (202), (310), (222), (312), (320), (213) and (004). All peaks can be matched with the HAP ICDD no. 09-0432. The absence of a peak at 29.5° indicates that calcite was not associated with HAP, and this result agrees with FTIR data [54]. The XRD patterns displayed evident similarities in their characteristic peaks. However, there no differences were observed from peak broadening.

### 3.3. SEM and EDX Results

**Figure 5:**
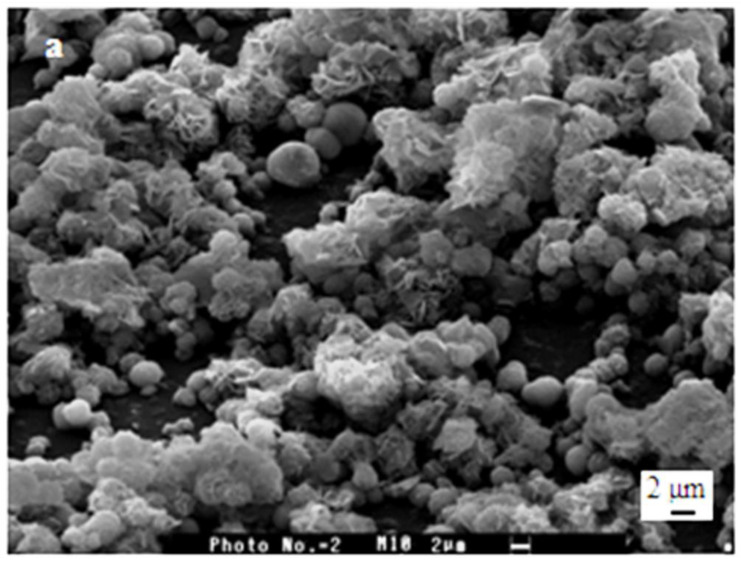

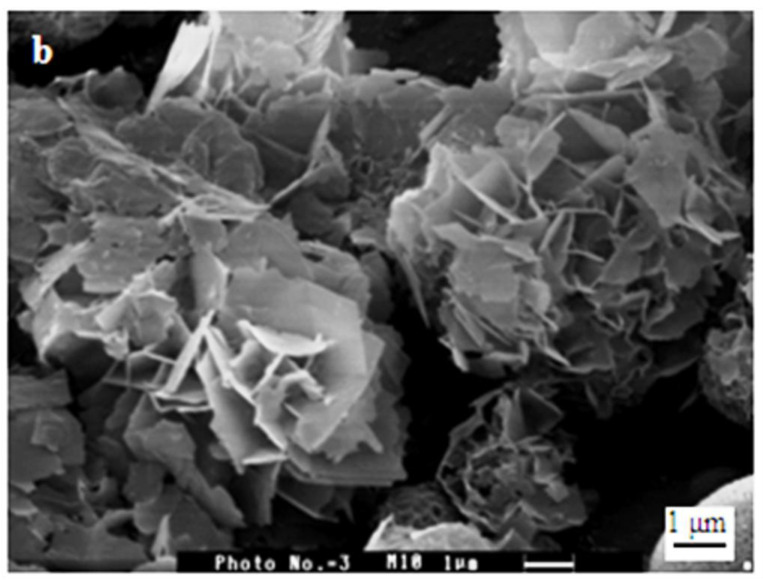
Scanning electron micrograph of a sample of PCS films after immersion in saturated Ca(OH)_2_ for eight days at room temperature. Scale bars: (**a**) 2 μm, (**b**) 1 μm.

Figure 5 shows SEM photos of the PCS film surface after immersion in Ca(OH)_2_ solution for eight days at room temperature.

Radiated petal-like crystals assembled into flower-like clusters with sizes in the range of 3 to 10 μm were observed by SEM on the surface of PCS films. This observation is confirmed by the results of Petrakova et al. [55].

A calcium phosphate layer was deposed when the Ca(OH)_2_ treated PCS films were immersed in an ASS solution. It can be supposed that the thin CaP coatings on the films, obtained after the partial hydrolysis of the PCS-PO_4_ groups during the immersion in saturated Ca(OH)_2_ solution, act as a nucleation layer upon which the crystals can grow from the ASS solution after one day. This indicates that, after the introduction of films into the ASS solution, the OCP partially dissolved. After 1–6 days of immersion, the CaP layer grows in number and size on the surface as circular flakes. A further increase in the thickness of the coating was observed after 28 days of soaking.

Ca(OH)_2_ treated PCS films were immersed in ASS solution as a function of time. After four days of immersion, a single layer of calcium phosphate was formed on the surface of the films. After eight days, CaP particles almost covered the surface, and this phenomenon coincided with secondary nucleation leading to a porous coating. Within 28 days, the entire surface of the films was coated with the porous layer of CaP.

**Figure 6:**
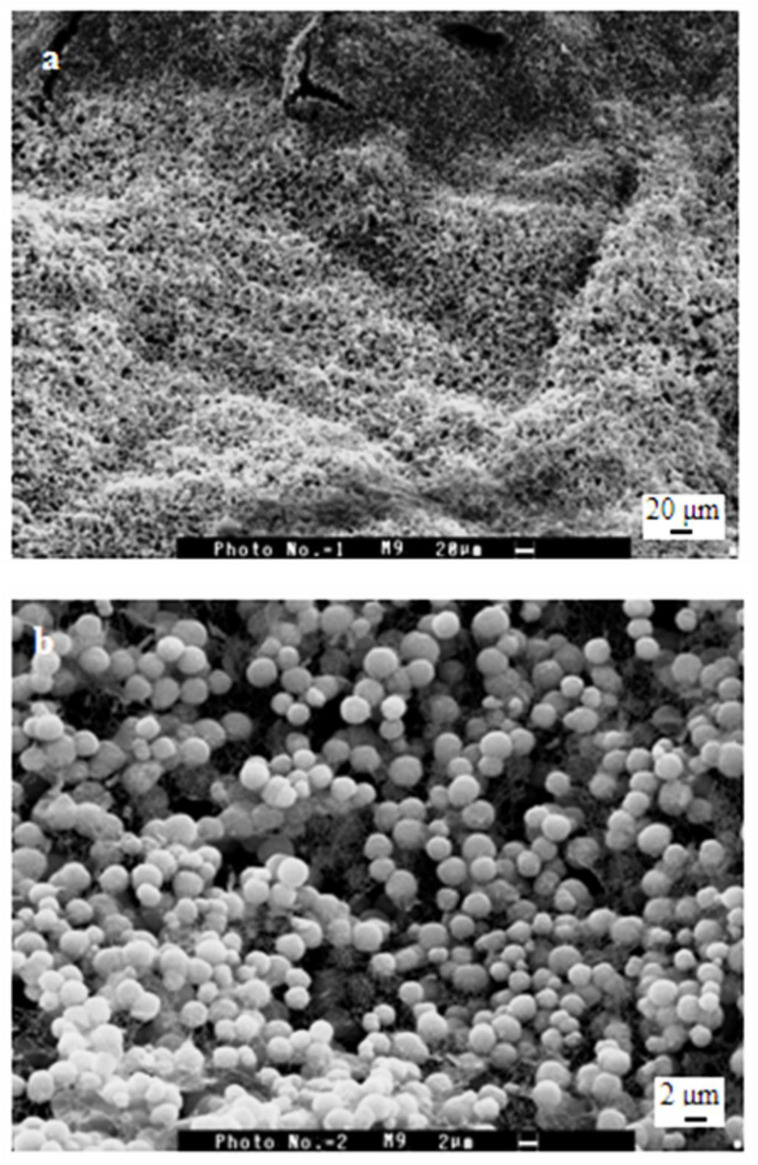

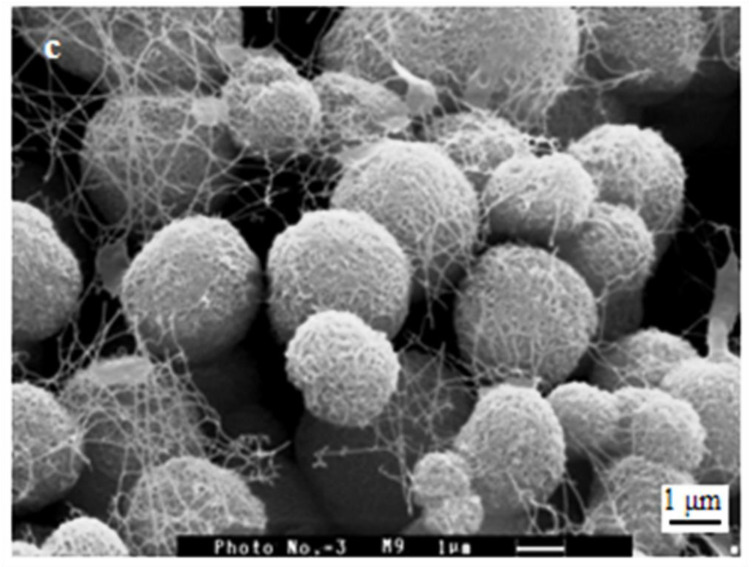
Scanning electron micrograph of samples of Ca(OH)_2_ treated PCS films after soaking in ASS for 15 days. Scale bars: (**a**) 20 μm, (**b**) 2 μm, (**c**) 1 μm.

SEM micrograph of the analyzed immersed materials in ASS proves the evident dissolution of OCP crystals (Figure 6). In addition, after four days of immersion, thinner and tilt petal-like OCP crystals with irregular faces were noticed.

**Figure 7:**
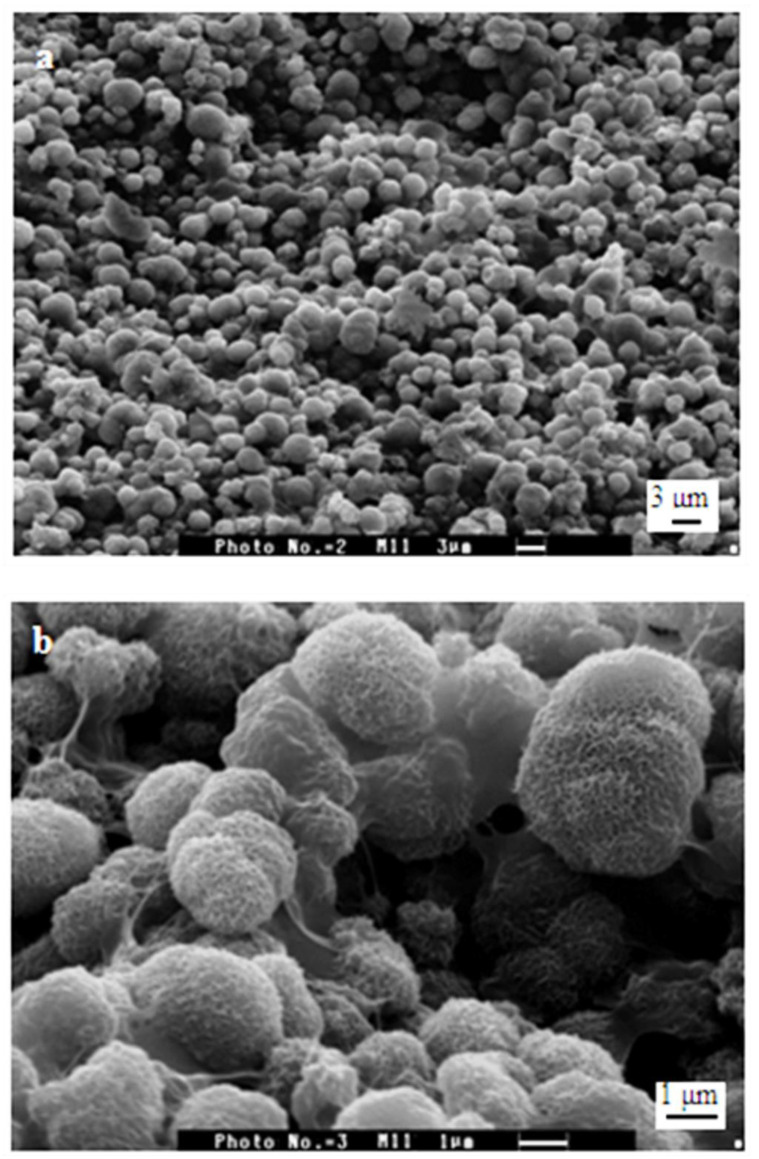
Scanning electron micrograph of samples of Ca(OH)_2_ treated PCS films after 28 days immersion in ASS. Scale bars: (**a**) 3 μm, (**b**) 1 μm.

On the 28th day of soaking (Figure 7), a thin amorphous layer was noticed on almost the film’s surface. The plate-like crystals are now smaller, porous, and rough with sharp points [55].

The Ca/P ratio is commonly used in discussions of phases of calcium phosphates. The change in Ca/P influences biomineralization. It affects the structure of calcium phosphate deposit material, shape, and size. It also affects the chemistry of the material. The Ca/P changes as a function of the time of in vitro biomineralization in ASS. The increase in the Ca/P ratio provides information on the nature of the calcium phosphate phase deposited on the surface of the films, the Ca/P increases, and the calcium phosphate phase OCP is transformed into HAP.

EDX tests, given in Figure S2, illustrate the Ca/P ratio for the CaP coating formed on Ca(OH)_2_ treated PCS films as a function of immersion time in ASS. Ca/P ratios, determined at the surface of films, ranged from 1.28 to 1.55. As a function of time, the Ca/P molar ratio increases in the mineral crystals from the bone. The supersaturation concerning HAP in the tissue fluid is the driving force which induces the nucleation and propagation of bone crystals within the matrix. OCP and HAP’s theoretical Ca/P molar ratios are 1.33 and 1.67, respectively [43].

**Figure 8:**
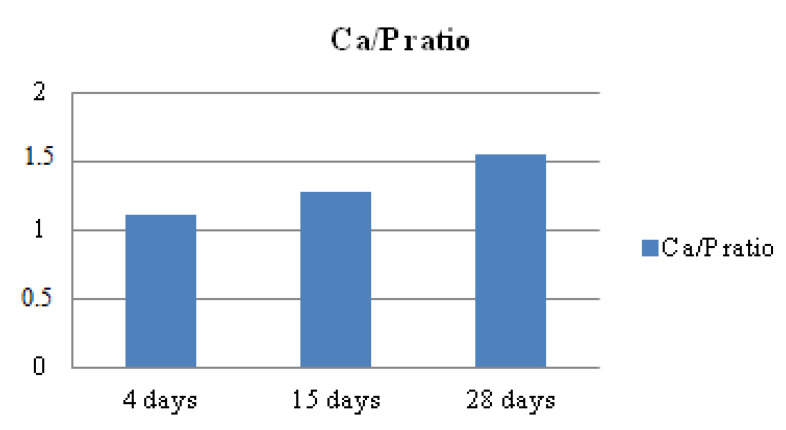
Ca/P ratio for the CaP coating formed on Ca(OH)_2_ treated PCS films as a function of immersion time in ASS solution.

EDX spectra show that the coating layer obtained after immersion in ASS during 4, 15 and 28 days is majority formed by Ca and P molecules. As the immersion time increases, the Ca/P ratio also increases to 1.55, close to the theoretical value of 1.67, characteristic of HAP. This evolution is provided in Figure 8.

It should be noted that the variation in the Ca/P ratios obtained by the XPS and EDX techniques is because XP is a technique that aims at surface analysis. It has been used to determine the chemical composition of the surface of films before and after biomineralization. In contrast, the EDX analysis was used to determine the bulk elemental composition present in the films after biomineralization, especially the calcium and phosphate, to determine subsequently the Ca/P ratios, ranging from 1.28 to 1.55.

### 3.4. XPS Results

**Figure 9:**
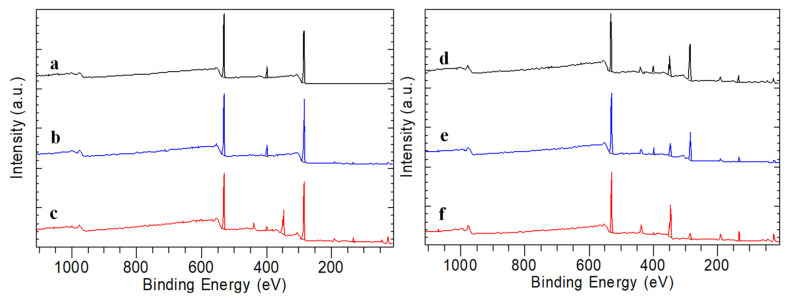
Typical XPS spectra of (a) CS films (b) PCS films (c) PCS-Ca(OH)_2_ treated films (d) PCS-Ca(OH)_2_ treated four days biomineralized (e) PCS-Ca(OH)_2_ treated 15 days biomineralized (f) PCS-Ca(OH)_2_ treated 28 days biomineralized.

The presence of carbon, oxygen and nitrogen can be confirmed in the XPS spectra of CS films, as illustrated in Figure 9. After the phosphorylation reaction, phosphorus was detected on all films; also calcium was detected in biomineralized films in ASS. The presence of other atoms was observed, as expected.

The concentration of Ca, P and O in the PCS-Ca(OH)_2_-treated 28 days biomineralized was lower than the stoichiometric values. From the atomic concentration (%) values, the Ca/P ratios were calculated, and they ranged from 1.13 to 2.33. The Ca(OH)_2_ treated PCS films (before immersion in ASS) have a Ca/P ratio of 2.33 (Table 2), indicating that the surface has a high amount of Ca.

From Table 2, it appears, for most samples, that soaking in ASS subsequently leads to a reduction of the Ca/P ratio. Nevertheless, the coating layer has a high amount of Ca. Even if this value is lower than 1.67, the theoretical Ca/P ratio for stoichiometric HAP is acceptable since the HAP surface can be calcium deficient. It is well known that the bulk Ca/P ratio varies from 1.3 to 2.0 in concordance with HAP’s phase and composition [56].

Moreover, according to quantitative analysis, the relative surface compositions of different elements indicate that with the biomineralization time, the relative atomic contents of P, O and Ca raised to 11.93, 47.84 and 16.33%, respectively. By contrast, C and N decreased to 18.85 and 1.28%, respectively. Table 3 provides the experimental XPS and theoretical values of different molar ratios for CS and PCS.

Table 3 shows that the majority of the experimental values are in good agreement with the theoretical ones. However, the ratio of the C_284.8_/C component is much higher than expected, indicating the presence of adventitious organic contaminants, characteristic of solids with a high surface energy [57].

By considering atomic element percentages, CS films revealed the presence of a higher amount of carbon, whereas oxygen and nitrogen are observed in a smaller amount. Nevertheless, in the case of the O/N ratio, a value of 4.643, close to the expected one, was calculated for CS as phosphorus atomic percentage is correlated with the amount of nitrogen and considering that each CS sequence has one nitrogen atom, a direct relation can be established between the P/N atomic ratio and the degree of substitution at the surface.

As expected, the concentration of oxygen increases with the reaction time. As a result, three new oxygen atoms are formed for each grafted phosphate group. Using Equation (2) [31], it was possible to calculate the expected values by normalizing the element surface concentrations to nitrogen content.

(2) $$\frac{O}{P}=\frac{4.643+(3\times \frac{P}{N})}{P/N}$$

**Figure 10:**
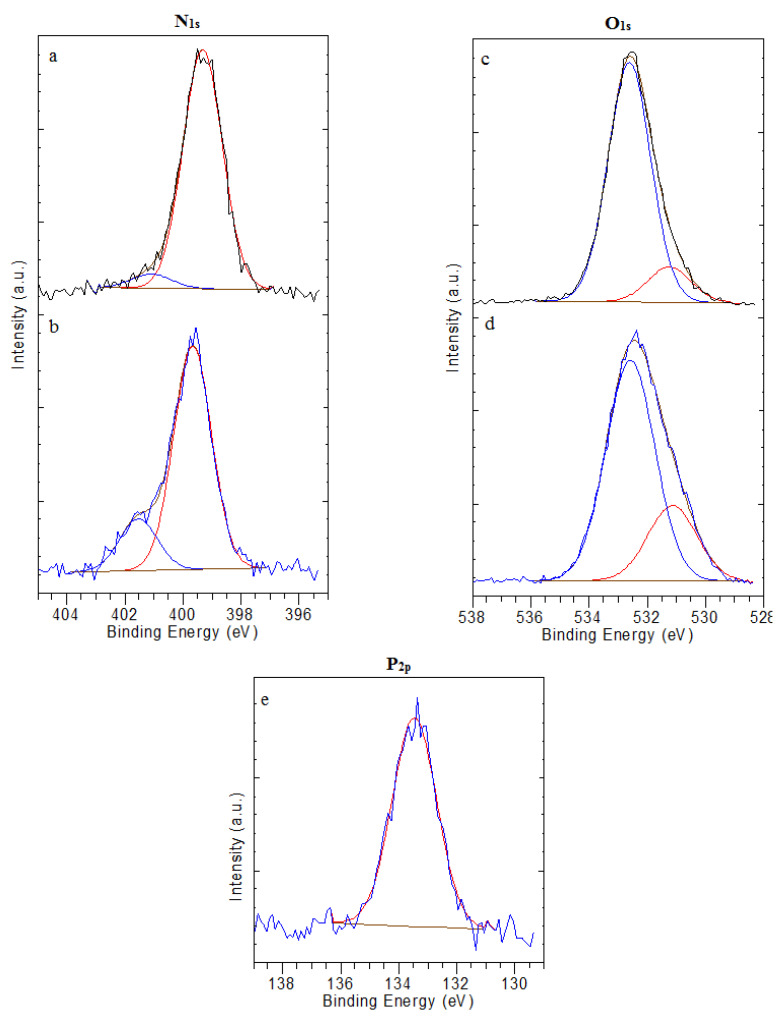
N_1S_, O_1S_ and P_2P_ XPS high-resolution spectra of: (**a**,**c**) unmodified CS films, (**b**,**d**,**e**) PCS films.

Figure 10a shows the spectrum N_1S_ of the unmodified CS films. In this spectrum, the peak identified at 399.3 eV is assigned either to non-protonated amine or to the amide NH_2_ and N−C=O chemical bindings [31]. The small peak observed at 401.1 eV is assigned to the protonated amine NH_3_^+^.The amount of nitrogen atoms increases as a consequence of the phosphorylation reaction. Moreover, the intensity of the N1S component, characteristic of NH_2_ and N−C=O chemical bindings and observed at 399.7 eV, is decreased (Figure 10b). This behavior might be explained by the low pH value of the reaction medium. Protonated amines, in the form of ammonium salts, are present in both phosphate groups substituted to the chains of CS or in free phosphates. This presence is demonstrated by the shift of the electron binding energy (0.4 eV) of the NH_3_^+^ peak at 401.5 eV [31,42].

The O_1S_ spectrum of unmodified CS and PCS are given in Figure 10c,d. In the spectrum of unmodified CS (Figure 10c), two peaks are observed. The peak at 531.2 eV is attributed to the amide of the acetylated functions (N−C=O in N-acetylated-glucosamine units). In contrast, the peak at 532.6 eV is assigned to the C−O, C−OH and O−C−O chemical bindings from the CS and PCS backbone [57].

The successful deposition of the phosphorus layer on the CS films is demonstrated by elemental phosphorus peaks (Figure 10e). As in the spectra from PCS films, the binding energy peak at 532.6 eV for O_1S_ in C−OH did not change or shift, confirming that the phosphorylation reaction does not occur in the hydroxyl group.

**Figure 11:**
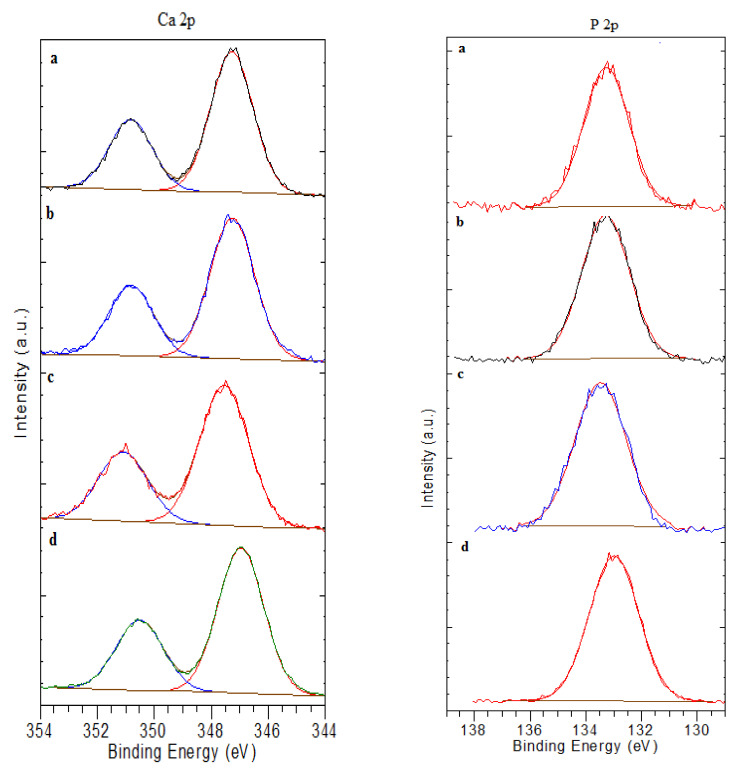
Ca_2P_ and P_2P_ XPS high-resolution spectra of (**a**) PCS-Ca(OH)_2_ treated (**b**) PCS-Ca(OH)_2_ treated four days biomineralized (**c**) PCS-Ca(OH)_2_ treated 15 days biomineralized (**d**) PCS-Ca(OH)_2_ treated 28 days biomineralized.

The phosphate ester has two pKa values for the phosphate group, which should be mono-hydrogen phosphate −HPO_4_^2−^ at neutral pH. Thus, the binding energy of the peak is noticed at 531.8 eV. Amaral et al. [31] demonstrated that the peak at 531.2 eV is assigned to the binding energy of H_2_PO_4_^−^ groups from the protonated amine groups. Moreover, the peak at 531.2 eV is assigned to N−C=O, O−C−O, P−OH and P=O chemical bindings. In the spectra of PCS-(CaOH_2_) treated 28 days biomineralized in ASS (Figure 11b), two peaks are identified. The peak at 531.2 eV is assigned to the mineral oxygen P=O and P−O. The peak at 532.6 eV is attributed to the C=O and C−O organic oxygen.

Figure 10e shows the P_2P_ spectra. The spectrum from PCS films revealed a single peak centered at 133.5 eV, which was assigned to phosphate species such as: −HPO_4_^2^ and H_2_PO_4_^−^ (P−OH and P=O chemical bindings) [31]. All other spectra presented in Figure 11 related to P_2P_ XPS high resolution of PCS-Ca(OH)_2_ treated, PCS-Ca(OH)_2_ treated for four days biomineralized, PCS-Ca(OH)_2_ treated for 15 days biomineralized and PCS-Ca(OH)_2_ treated for 28 days biomineralized in ASS revealed a single peak centered in the range (133.0 to 133.5 eV). Those peaks are characteristic to P−OH and P=O groups.

Figure 11 illustrates the Ca_2P_ spectra. All the spectrum related to PCS-Ca(OH)_2_ treated, PCS-Ca(OH)_2_ treated four days biomineralized, PCS-Ca(OH)_2_ treated 15 days biomineralized and PCS-Ca(OH)_2_ treated 28 days biomineralized in ASS revealed two peaks assigned to Ca_2P 3/2_ and Ca_2P 1/2_ [57,58].

**Figure 12:**
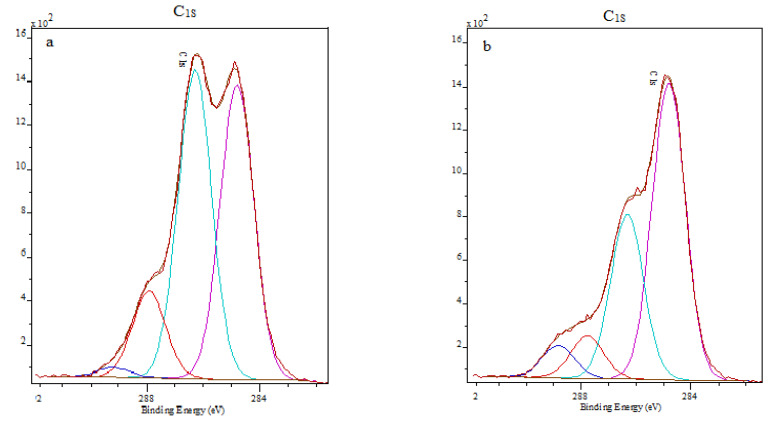
C_1S_ XPS high-resolution spectra of (**a**) unmodified CS films, (**b**) PCS films.

The results of the C_1S_ spectra of unmodified CS films and PCS films are presented in Figure 12.

The resolved C_1S_ spectrum of the CS films shows four peaks (Figure 12a). The C_1S_ peak at 284.8 eV is assigned to the carbon surface contaminant −CH_2_−, but also to the C−NH_2_ group. The peak at 286.3 eV is attributed to C−O, C−OH and C−N−C=O, whereas the peak at 287.9 eV is characteristic of O−C−O and N−C=O. Phosphorylated samples (Figure 12b) have similar XPS spectra. The peak at 288.8 eV (more specifically in the range 288.9 to 289.6 eV) is close to that of ester or carbonate, which will be referred to as the component (C_hibe_) [57,58]. The C_1S_ peak characteristic to C−O−P and expected at 286.4 eV, was probably overlapped by the C−OH group from CS, which appear normally at 286.3 eV [31]. In the spectrum of PCS-Ca(OH)_2_ biomineralized for 28 days in ASS (Figure S3), the C_1S_ peak at 284.8 eV is attributed to the carbon surface contaminant −CH_2_−, but also to the C−NH_2_ group. The peak at 286.3 eV is characteristic of C−O, C−OH and C−N−C=O, whereas the peak at 287.7 eV is assigned to O−C−O and N−C=O groups. The peak at 288.8 eV was assigned to CO_3_ (C=O and C−O), which is in concordance with FTIR and XRD results.

### 3.5. Weight Test Results

**Figure 13:**
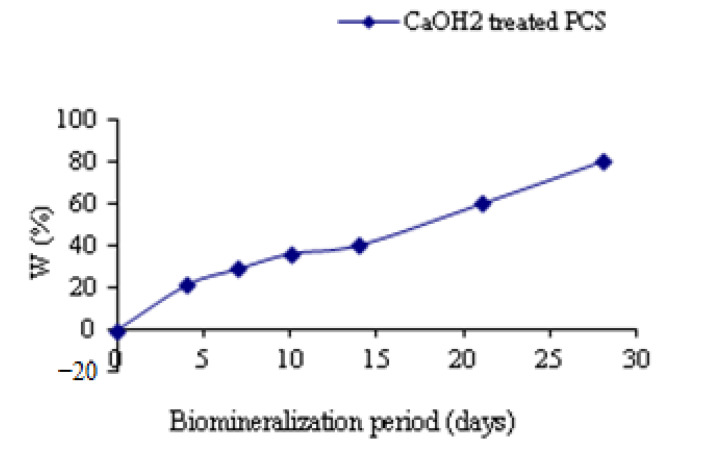
Weight variation of biomineralized films in ASS as a time function.

The comportment of the films in the ASS was determined by studying the variation of the weight of films versus the time of biomineralization in ASS (Figure 13). Recording to the obtained results, the weight increase of films as a function of biomineralization time was due to the deposition and growth of CaP layers on the surface of the film, which has confirmed that Ca(OH)_2_-treated PCS can promote the crystallization kinetics from ASS to form HAP.

### 3.6. Antimicrobial Activity of PCS

PCS showed good antibacterial activity against the microorganisms tested (Table 4). Furthermore, the antibacterial activity of the PCS films was found to be concentration dependent, whereas no activity was observed in the negative control.

The highest Minimum Inhibitory Concentrations (MIC) value was obtained for Candida Albicans, specific fungi found in the oral cavities.

## 4. Conclusions

The evaluation of the biomineralization process is important not only to have a deeper understanding of the in vivo obtaining of mineral-rich tissues but also because it clarifies the mechanism related to the design of advanced materials with possible application in dentistry.

The results of the present study proved that phosphorylation and partial hydrolysis in intimate contact with the substrate is a useful method for obtaining beneficial local conditions which will further allow the nucleation and growth of CaP (carbonate HAP).

I found that the phosphorylated CS films immersed in saturated Ca(OH)_2_ solution, at room temperature, induces the fast growth of a CaP coating on their surfaces, generally after the first day of soaking in ASS. The mechanism of coating formation involves the dissolution of the OCP after the introduction of the Ca(OH)_2_-treated PCS samples into the ASS. This process increases the Ca^2+^ ion concentration and creates a sufficiently high local concentration of ions at the film’s surface which will further facilitate the precipitation of CaP from the immersion medium.

Biomineralization was assessed by SEM/EDX, RDX, XPS and FTIR spectroscopy and confirmed the ability to promote the growth of carbonate HAP crystal through the biomimetic method in ASS with a Ca/P close of the stoichiometric HAP. Moreover, the PCS films have shown a concentration-dependent antimicrobial activity.

The proposed method is essential for designing complex biomaterials, combining the advantages of both inorganic and organic materials. In addition, this can help mimic the biomineralization process used in dental tissue regenerative medicine as dental substitute materials.

## Figures and Tables

**Table 1:** Ionic composition of natural and artificial saliva. [29].

Ions	Natural Saliva (mM/L)	Artificial Saliva (mM/L)
Ca^2+^	1.43	1.43
Mg^2+^	0.21	0.21
K^+^	22.7	20.50
Na^+^	10.13	11.55
CO_3_^2−^	6.45	6.45
CL^−^	23.22	23.22
PO_4_^3−^	5.24	5.10

**Table 2:** XPS data of PCS films, Ca(OH)_2_-treated PCS films (PCS-Ca(OH)_2_-treated) after biomineralization in ASS for 4, 14 and 28 days, respectively.

Samples	N (%)	C (%)	O (%)	Ca (%)	P (%)	Ca/P
PCS	5.82	67.89	24.64		1.52	
PCS-Ca(OH)_2_-treated	2.44	58.34	28.14	7.75	3.32	2.33
PCS-Ca(OH)_2_-treated four days biomineralized	4.07	51.95	31.88	6.65	5.45	1.22
PCS-Ca(OH)_2_-treated 15 days biomineralized	5.69	44.16	39.4	5.41	4.8	1.13
PCS-Ca(OH)_2_-treated 28 days biomineralized	1.28	18.85	47.84	16.33	11.93	1.37
Stoichiometric HAP			59.1	22.7	13.63	1.67

**Table 3:** Molar concentration ratios of CS and PCS were expected and deduced from XPS spectra.

Elements	CS		PCS	
	Expected	XPS	Expected	XPS
N/C_286.2_	0.2	0.195	0.2	0.280
(O+N)/C_286.2_	1.2	1.103	1.2	0.720
C_287.7_/C_286.2_	0.3	0.282	0.3	0.260
(O+N)/(C_286.3_+C_287.7_)	0.923	0.860	0.923	0.571
O/C_286.2_	1	0.908	1	0.624
O_532.6/_C_286.2_	0.9	0.792	0.9	0.881
N/C_287.7_	0.667	0.693	0.667	1.071
C_284.8_/C	0.071	0.420	0.071	0.553
O_532.6_/O_531.2_	9	6.851	2.25	2.92
O/N	5	4.643	6.5	4.234
O/P			13	16.21
O_531.2_/P			4	4.13

**Table 4:** Minimum Inhibitory Concentration (MIC) of PCS.

Microorganisms	MIC of PCS (%)
*Candida albicans*	0.10
*Staphylocoques aureus*	0.05
*Escherichia coli*	0.025

## Data Availability

Not applicable.

## Supplementary Materials

The following supporting information can be downloaded at: https://www.mdpi.com/article/10.3390/polym15112470/s1, Figure S1: XRD spectra of (a): unmodified CS and (b) PCS films.; Figure S2: Representative EDX spectra after soaking in ASS for (a) 4 days, (b) 15 days and (c) 28 days. Ca/P ratios of coated films were for: (a) 1.11 (1.13 found per XPS), (b) 1.28 (1.22 found per XPS) (c) 1.55 (1.37 found per XPS); Figure S3: C_1S_ (a) and O_1S_ (b) XPS high-resolution spectra of PCS-Ca(OH)_2_ treated 28 days biomineralized in ASS.
