# Supplementary material for: Biomimetic Hydroxyapatite Crystals Growth on Phosphorylated Chitosan Films by In Vitro Mineralization Used as Dental Substitute Materials
Source: Polymers (Basel). 2023 May 26;15(11):2470. doi: 10.3390/polym15112470 (PMC10255572; doi:10.3390/polym15112470)
Supplement: Supplementary file 1 [file polymers-15-02470-s001.zip › polymers-2358535-supplementary.pdf]

Supporting Information

Biomimetic Hydroxyapatite Crystals Growth on Phosphorylated Chitosan Films by *In Vitro* Mineralization Used as Dental Substitute Materials

*Fathia Rahmani [1, 2], Omar Larbi Bouamrane[3,4, *], Amina Ben Bouabdallah[2,5,6], Leonard I. Atanase[78,*], Abdelkader Hellal[4,9], Aurelian Nichita Apintiliesei[7]*

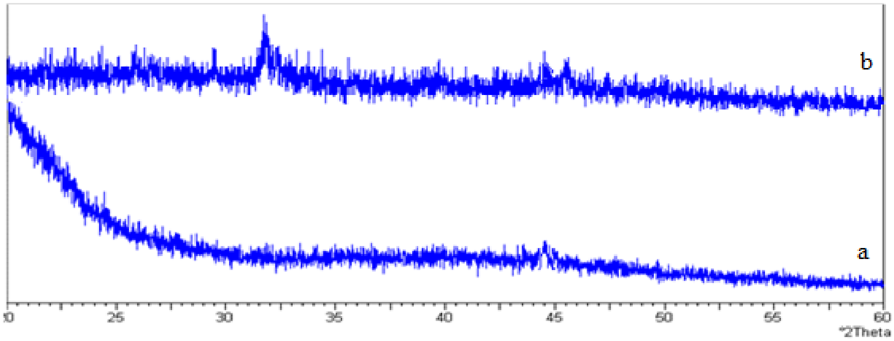

Figure S1: XRD spectra of (a): unmodified CS and (b) PCS films.

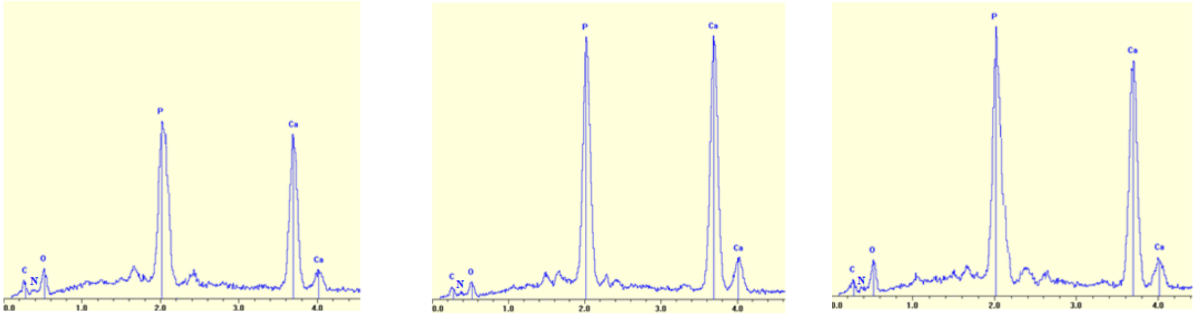

(a) (b) (c)

Figure S2: Representative EDX spectra after soaking in ASS for (a) 4 days, (b) 15 days and (c) 28 days. Ca/P ratios of coated films were for: (a) 1.11 (1.13 found per XPS), (b) 1.28 (1.22 found per XPS) (c) 1.55 (1.37 found per XPS).

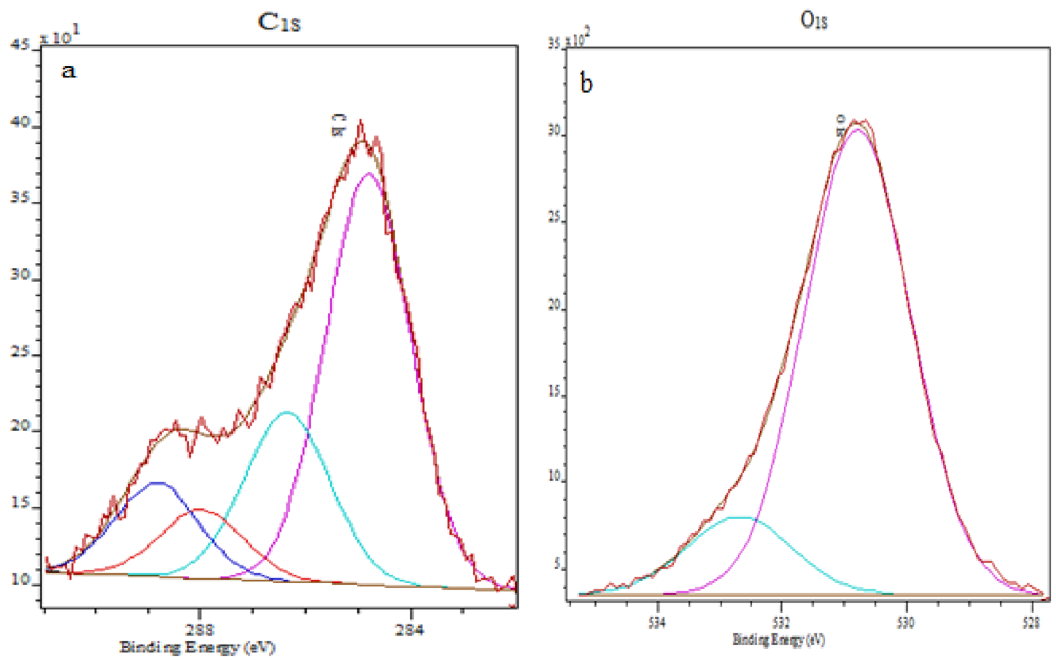

Figure S3: C_{1S} (a) and O_{1S} (b) XPS high resolution spectra of PCS-$Ca(OH)_2$ treated 28 days biomineralized in ASS.
